# Emerging technologies towards enhancing privacy in genomic data sharing

**DOI:** 10.1186/s13059-019-1741-0

**Published:** 2019-07-02

**Authors:** Bonnie Berger, Hyunghoon Cho

**Affiliations:** 1 Computer Science and Artificial Intelligence Laboratory, MIT, Cambridge, MA 02139 USA; 2 Department of Mathematics, MIT, Cambridge, MA 02139 USA; 3 Broad Institute of MIT and Harvard, Cambridge, MA 02142 USA

## Abstract

As the scale of genomic and health-related data explodes and our understanding of these data matures, the privacy of the individuals behind the data is increasingly at stake. Traditional approaches to protect privacy have fundamental limitations. Here we discuss emerging privacy-enhancing technologies that can enable broader data sharing and collaboration in genomics research.

## Promises of the genomic data deluge and potential for privacy leaks

As we enter the era of personalized medicine, large-scale datasets containing individual-level genomic and clinical data are becoming increasingly valuable to researchers. Analyzing data from a large, diverse study cohort is key to detecting fine-grain biological insights essential to improving public health. The pressing need for ‘big data’ in genomic medicine has long been recognized by the biomedical community, which has recently led to several large-scale genomic data collection efforts, including the All of Us Research Program in the United States [1] and the UK Biobank [2]. These efforts are resulting in biomedical datasets of unprecedented scale that will enable researchers to push the frontiers of genomic medicine.

With the growing scale of patient data in scientific studies, ensuring the privacy of study participants is becoming ever more important. A single data breach can now leak genomic and other health-related information on millions of individuals. These leaks may put the affected individuals at risk for genetic discrimination in employment or insurance (even if it is illegal), or unwanted disclosure of their biological family, medical history, or sensitive disease status. The scope of such harm could easily extend to descendants or relatives of the affected individuals as they share much of their genetic biology. Moreover, unlike user accounts and passwords (which are routinely leaked from IT companies), one’s genetic information cannot be changed at will—once it is leaked, it stays leaked.

## Traditional approaches towards protecting privacy and their limitations

Traditional approaches to protect the privacy of study participants in biomedical research often provide inadequate privacy guarantees in practice. The Health Insurance Portability and Accountability Act of 1996 (HIPAA)—one of the most prominent legal standards for biomedical research to this day—provides a guideline for handling sensitive patient data based on the technique of ‘de-identification’, which refers to the process of censoring or transforming the data so that the resulting data cannot be linked to the individual who provided it. Unfortunately, most de-identification techniques fail to guard against sophisticated re-identification attacks that exploit the data in an unforeseen manner. For example, an attacker may use an external database that shares a subset of data fields with the de-identified data to infer additional facts about the individuals and to subsequently uncover their identity. This is known as a ‘linkage’ attack. Sweeney [3] used this technique to combine a supposedly de-identified database of hospital records with a voter registration database to link a particular patient profile to the then-Governor of Massachusetts, demonstrating that de-identification, though useful as a minimal requirement, is not a guarantee for privacy. Notably, the General Data Protection Regulation (GDPR) 2016/679, recently implemented by the European Union, recognizes different levels of de-identification and introduces a weaker notion of de-identification called ‘pseudonymization’, which entails the removal of only the directly identifying information.

What makes privacy protection an especially challenging pursuit in genomics research is that fully de-identifying a genomic dataset while retaining its utility for research is likely not possible. A personal genome is unique to each individual (with the exception of twins), and a small number of genetic variants is enough to pinpoint an individual. A recent study showed that a person’s genotype profile can be queried against publicly accessible genealogical databases to reveal their identity through their relatives in the database [4]. It is worth noting that *functional* genomic data, such as transcriptomic or epigenomic read datasets, can also reveal the genetic variants of an individual [5]. Even if only the preprocessed functional measurements (e.g., transcript abundance) are shared, some of the underlying genotypes may be indirectly revealed through statistical associations known as quantitative trait loci (QTL). Given the growing importance of integrative studies that jointly consider a range of genomics experiments and clinical data from patients, the fact that genomic information is especially prone to re-identification attacks presents a pressing challenge for sharing these multi-modal datasets.

Another common strategy for reducing the privacy risks of biomedical data is ‘access control’, whereby data access is granted to a carefully chosen group of researchers. Most genomic data repositories, including the NIH NCBI’s database of Genotypes and Phenotypes (dbGaP) and the UK Biobank, require researchers to submit a summary of their proposed research, which is reviewed by a data access review committee to determine whether the project is within the scope of the informed consent given by the study participants. This process often takes many months. Although this gives study participants and biobanks finer control over *who* can access their data and for *what purpose* (a hallmark of privacy), it substantially limits the scope of data sharing, e.g., to researchers studying particular diseases or those within a particular organization, and does not alleviate concerns about a potential leakage once researchers obtain these data.

## Cryptographic approaches and their challenges

Recently developed theoretical frameworks from cryptography may provide alternative paradigms for sharing sensitive biomedical data with enhanced privacy protection. For example, secure multiparty computation (MPC) frameworks [6] allow multiple entities (e.g., research labs or regulatory agencies) to cooperatively carry out computational analyses while keeping the input data private. No involved entity—even the researchers performing the analyses—gains any information about the input data, other than what is revealed in the final output. Such a framework could facilitate collaboration across multiple institutes, where they pool their data for joint analyses while keeping the data private to the respective owners. This framework could also lead to new experimental designs with end-to-end data privacy. In this scenario, private data collected from patients is securely shared with a *group* of labs such that no single entity is entrusted with the raw data throughout the study. This enhanced privacy guarantee may broaden the scope of data sharing and enable collaborations that are currently not feasible due to regulatory constraints.

Other related technologies for enabling secure genomic analysis workflows include homomorphic encryption (HE) [7] and secure hardware-based approaches. HE provides a mechanism to encrypt data in a way that allows calculations to be performed over the underlying private numbers *implicitly* via operations over the encrypted dataset. Unlike MPC, HE requires only a single entity to perform the computation, which considerably simplifies the setup compared to MPC, albeit with significantly greater computational overhead using existing techniques. In a hardware-based approach, sensitive data are decrypted and analyzed only inside an isolated hardware environment called a ‘secure enclave’ (e.g., Intel Software Guard Extension, SGX), which keeps the data hidden from the user and other processes on the machine. Compared to cryptographic approaches such as MPC and HE, hardware-based approaches incur the least computational overhead as the main computation is performed over cleartext (unencrypted) data. Yet there are notable limitations of the approach, including limited memory capacity of the enclave and the lack of theoretical privacy guarantees—in fact, several security attacks on SGX have been demonstrated in the literature.

Given the tradeoffs between these related technologies, different study setups may call for different approaches for privacy protection to be employed. Currently, HE is best-suited for low-complexity analyses (e.g., calculating aggregate statistics) and is especially effective for settings where communication between the parties is costly. Alternatively, MPC addresses a wider range of analyses (e.g., principal component analysis [8] or neural network training [9]) by efficiently handling more complex computations at the cost of a higher communication burden. While SGX nearly matches the flexibility of analysis without privacy, except for a low-memory footprint requirement, it is limited to study settings where its weaker privacy guarantees can be considered sufficient.

Although the aforementioned cryptographic approaches allow researchers to analyze data without having direct access to the raw data, these tools do not address the potential leakage of sensitive information in the final results of computational analyses (e.g., aggregate statistics). It has been demonstrated that even coarse-level information such as minor allele frequencies (MAF) can reveal whether a given individual is part of the study cohort, potentially disclosing sensitive clinical phenotypes of the individual [10]. Differential privacy (DP) frameworks [11] may help address this concern by providing principled mechanisms for limiting the privacy leakage through adding a controlled amount of noise to the data. It is worth noting that the theoretical privacy guarantee of DP holds even in a linkage attack scenario where the attacker has access to external information. DP techniques cannot only be used to add another layer of privacy protection to secure computation pipelines, they can also help enhance privacy in interactive biomedical database services. Here researchers submit analysis queries and receive answers in a privacy-preserving manner through DP mechanisms.

## Challenges and future outlook

Despite the promises of emerging privacy-enhancing technologies, key hurdles remain for these tools to be widely adopted by the genomics community. The foremost challenge is that of scalability. Most existing frameworks for secure computation incur significant computational overhead for large-scale and complex data analysis tasks, which are common in biomedical data analysis. This limitation compels researchers to rely on small-scale datasets or simplified versions of the analysis tasks, which significantly limit the applicability of privacy-preserving techniques. Although recent advances from our group [8, 9] and others [12] present a path towards scalable secure pipelines for key analysis tasks in the field such as genome-wide association studies (GWAS), most data analysis workflows in biomedicine currently lack privacy-preserving alternatives that scale to real-world settings. Differential privacy frameworks face similar challenges for practical adoption; existing techniques often require excessive amounts of noise to be added when applied to large-scale data releases (e.g., association statistics at genome-scale). Community-wide efforts for methodological development such as the iDASH Secure Genome Analysis competition [12] will be increasingly important as the needs for privacy-enhancing methods in the field continue to grow.

Another challenge is navigating the complex landscape of policies and regulations to drive the incorporation of privacy-preserving technologies. Since most existing regulatory frameworks are designed for the sharing of *cleartext* data, creating the capacity for and defining the limits of new workflows based on the emerging privacy-preserving technologies require new laws and policy guidelines. Given the varying requirements and privacy guarantees of these technologies, many of which are still under active development, efforts to standardize the use of these frameworks in biomedical research will be immensely valuable for new policy development. International standard-setting organizations for genomics research pipelines, such as the Global Alliance for Genomics and Health (GA4GH) and the MPEG-G Consortium, may be well-positioned to play a pivotal role in this regard.

Preventive measures to mitigate privacy risks in biomedicine are sometimes regarded as a nuisance in scientific research, limiting researchers’ access to data. However, often overlooked is the widely liberating aspect of privacy-preserving technologies. Akin to how anonymity and privacy in the age of the Internet have provided a foundation for freedom of expression and increased visibility of minority groups, systems that enable the sharing of biomedical data with privacy may unlock a new wave of scientific studies that bridge the gap across nations, organizations, and communities to accelerate and promote inclusivity in future genomics research.

## Abbreviations

DP: Differential privacy
HE: Homomorphic encryption
MPC: Multiparty computation
SGX: Intel software guard extension

## References

[1] Sankar PL, Parker LS (2017). The precision medicine Initiative’s all of us research program: an agenda for research on its ethical, legal, and social issues. Genet Med.

[2] Sudlow C, Gallacher J, Allen N, Beral V, Burton P, Danesh J (2015). UK biobank: an open access resource for identifying the causes of a wide range of complex diseases of middle and old age. PLoS Med.

[3] Sweeney L (2002). K-anonymity: a model for protecting privacy. Int J Uncertainty Fuzziness Knowl-Based Syst.

[4] Erlich Y (2018). Identity inference of genomic data using long-range familial searches. Science..

[5] Harmanci A, Gerstein M (2018). Analysis of sensitive information leakage in functional genomics signal profiles through genomic deletions. Nat Commun.

[6] Cramer R, Damgård IB, Nielsen JB (2015). Secure multiparty computation.

[7] Gentry C, Boneh D (2009). A fully homomorphic encryption scheme, vol 20, no 9.

[8] Cho H, Wu DJ, Berger B (2018). Secure genome-wide association analysis using multiparty computation. Nat Biotechnol.

[9] Hie B, Cho H, Berger B (2018). Realizing private and practical pharmacological collaboration. Science..

[10] Homer N, Szelinger S, Redman M, Duggan D, Tembe W, Muehling J (2008). Resolving individuals contributing trace amounts of DNA to highly complex mixtures using high-density SNP genotyping microarrays. PLoS Genet.

[11] Dwork C, Roth A (2014). The algorithmic foundations of differential privacy. Found Trends Theoretical Comput Sci.

[12] Wang X, Tang H, Wang S, Jiang X, Wang W, Bu D (2018). iDASH secure genome analysis competition 2017. BMC Med Genet.

